# Maltol Protects Neuronal Cells by Alleviating Chronic Neuroinflammation, Pyroptosis, and Ferroptosis via HSP70 Upregulation in Microglia

**DOI:** 10.3390/nu18132071

**Published:** 2026-06-24

**Authors:** Jian-Qiang Wang, Bing-Bing Hu, Yi-Yue Wang, Ya-Wei Lu, Xiao-Jie Gong, Shan Tang, Ling-Jie Song, Yin-Shi Sun, Jing-Tian Zhang, Zi Wang, Wei Li

**Affiliations:** 1 College of Chinese Medicinal Materials, Jilin Provincial International Joint Research Center for the Development and Utilization of Authentic Medicinal Materials, Jilin Agricultural University, Changchun 130118, Chinazjt18104440010@163.com (J.-T.Z.); wangzi8020@126.com (Z.W.); 2 Jilin Provincial Center for Drug Review and Inspection (Jilin Provincial Center for Vaccine Inspection), Changchun 130022, China; 3 College of Life Sciences, Jilin Agricultural University, Changchun 130118, China; 4 Department of Biological Engineering, College of Life Science, Dalian Minzu University, Dalian 116600, China; 5 Institute of Special Animal and Plant Sciences, Chinese Academy of Agricultural Sciences, Changchun 130112, China

**Keywords:** maltol, ginseng, neuroinflammation, microglial pyroptosis, HSP70, HMC3 cells

## Abstract

**Objectives:** Neuroinflammation is recognized as a significant characteristic of Alzheimer’s disease (AD). Currently, there is a notable absence of effective pharmacological agents to prevent or treat neuroinflammatory processes associated with AD. Heat shock protein 70 (HSP70) is pivotal in the progression of neuroinflammation. In this study, we explored the potential of maltol, a Maillard reaction product derived from red ginseng, as a therapeutic agent for neuroinflammation. **Methods:** In vitro, HMC3 microglial cell models were developed to examine the regulatory effects of gradient concentrations of maltol (12.5, 25, 50 μM) on the TLR4/MyD88/NF-κB p65 signaling pathway, neuroinflammation, and pyroptosis. Analyses of the GEO database and Gene Set Enrichment Analysis (GSEA) were performed to identify the core targets of maltol, followed by HSP70 gene silencing experiments to validate the targeted regulatory mechanism. **Results:** Maltol significantly mitigated LPS-induced neuronal damage and cognitive deficits in mice. It effectively suppressed microglia-mediated neuroinflammation and pyroptosis, reversed oxidative stress-induced neuronal ferroptosis, and inhibited neuronal apoptosis. In vitro experiments demonstrated that maltol obstructed TLR4/MyD88 binding, thereby inhibiting NF-κB p65-mediated neuroinflammation and pyroptosis, while also alleviating excessive ROS accumulation to enhance oxidative stress and ferroptosis. Bioinformatics analysis identified HSP70 as a crucial target for the anti-inflammatory and antioxidant effects of maltol. Subsequent gene silencing experiments confirmed that maltol exerted its inhibitory effects on LPS-induced neuroinflammation and pyroptosis in an HSP70-dependent manner. **Conclusions:** Maltol exhibits significant protective effects against Alzheimer’s disease-related neuroinflammation, oxidative stress, pyroptosis, and ferroptosis through the targeting of HSP70. This study elucidates the molecular mechanisms by which maltol improves neuroinflammatory injury and provides a novel theoretical foundation and therapeutic strategy for the intervention of Alzheimer’s disease neuroinflammation using traditional Chinese medicine.

## 1. Introduction

Alzheimer’s disease (AD) is an irreversible neurodegenerative condition characterized by the accumulation of extracellular amyloid β (Aβ) peptide and intracellular Tau protein [1]. Neuroinflammation is a key factor in the development of AD [2], interacting with amyloid pathology to promote Tau formation and spread, ultimately causing neuronal apoptosis and cognitive decline [2,3]. Microglia, specialized macrophages within the brain, have a multifaceted impact on brain function and development. The neuroinflammatory response and neuronal harm orchestrated by micrBVCXoglia are closely linked to cognitive deficits [4].

Recent evidence indicates that microglial pyroptosis significantly contributes to neuroinflammation. It is widely recognized that aging, neuroinflammation, and the excessive production of reactive oxygen species (ROS) can largely result in oxidative stress and apoptosis of neuronal cells [5,6], which are inextricably linked. Intracellular and intercellular signaling and environmental stress can influence ferroptosis by regulating cellular metabolism and ROS levels [7]. Ferroptosis, a distinctive form of cell demise, is characterized by iron-mediated phospholipid oxidation and is influenced by diverse cellular metabolic routes. Numerous instances of organ damage and degenerative ailments stem from ferroptosis [8]. Therefore, exploring and pharmacologically regulating various metabolic pathways to reduce neuronal damage has great potential in the treatment of neuroinflammation.

Heat shock proteins (HSPs) are proteins synthesized by organisms in response to physical, chemical, biological, and mental stimuli in the environment [9]. The HSP70 protein is frequently upregulated in nearly all cells experiencing biological stress and plays a crucial role in numerous protective functions within the body. Its biological functions are extensive; under various stress stimuli, the synthesis of HSP70 enhances the tolerance of stressed cells and accelerates the restoration of normal protein synthesis [9], so that the reduction caused by protein denaturation can be supplemented and play the role of molecular chaperone. In addition, the increased expression level of intracellular HSP70 genes could inhibit the apoptosis and oxidative stress of inflammatory cells [10] to have an anti-apoptosis and antioxidative stress effect. Recent studies have revealed that elevated levels of HSP70 can uphold immune system equilibrium by suppressing NLRP3 activation and the ensuing inflammatory reactions in cardiomyocytes [11]. This underscores the potential of HSP70 as a target for enhancing immune modulation, prompting a burgeoning interest in exploring novel therapeutic agents aimed at this target.

Maltol (3-hydroxy-2-methyl-4-pyrone) is a naturally occurring pyran compound and a widely used food flavoring agent. It exhibits remarkable physical and chemical properties, demonstrating a strong affinity for trace metal ions. This compound is prevalent in various natural plants, including larch, fir, licorice, sweet birch, and magnolia. Additionally, maltol is a byproduct formed during the steaming and roasting of fresh ginseng (*Panax ginseng* C.A. Meyer) to produce red ginseng [12]. Our previous studies have shown that maltol could delay brain aging [13] and may have a good protective effect on the gut [14], liver, and kidneys [15], especially in terms of antioxidant and anti-apoptosis. There is little literature on the anti-inflammatory effects of maltol, and previous studies have shown that maltol improved intervertebral disc degeneration [16] and acetaminophen-induced hepatotoxicity by inhibiting the PI3K/AKT/NF-κB p65 pathway [17]. Donepezil has received broad approval from the US Food and Drug Administration (FDA) for the clinical treatment of AD [18]. Due to its efficacy in enhancing memory and learning, it is frequently employed as a positive control in clinical trials. Current research indicates that donepezil may ameliorate lipopolysaccharide (LPS)-induced neuroinflammation via the MAPK/NLRP3/STAT3 signaling pathway [19].

Drawing on maltol’s strong biological activity and well-established research foundation, we established in vitro and in vivo LPS-induced neuroinflammation models to investigate maltol’s potential protective effects against chronic neuroinflammation and elucidate its underlying molecular mechanisms. These results will serve as a crucial theoretical framework for advancing research aimed at ameliorating chronic neuroinflammation.

## 2. Materials and Methods

### 2.1. Chemicals and Reagents

**Figure 1:**
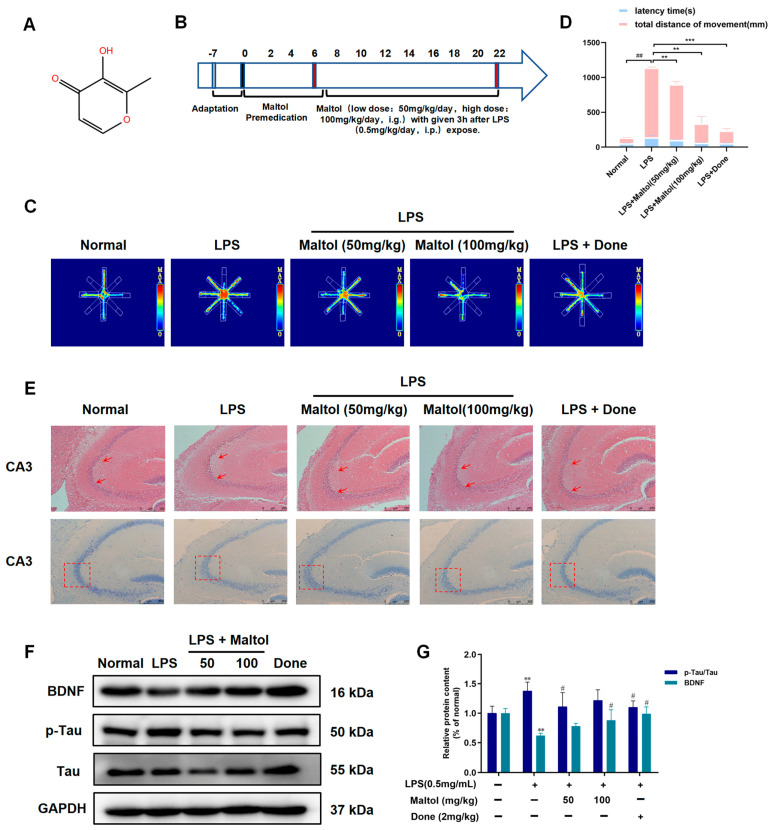
Maltol improved memory loss and ameliorated AD-like symptoms in vivo. (**A**) The chemical structure of maltol. (**B**) Animal experiments design. (**C**,**D**) Radial arm maze behavioral test. (**E**) H&E and Nissl staining of hippocampal CA3 regions (magnification, 200×). (**F**) Effect of maltol on the expression of proteins characterizing AD caused by LPS. (G) Histogram analysis of expressions of BDNF, p-Tau, and Tau proteins. Data are expressed as Mean ± S.D. (n = 8 in each group). # *p* < 0.05, ## *p* < 0.01 vs. normal group; ** *p* < 0.01, *** *p* < 0.001 vs. LPS group.

Maltol was purchased from Aladdin Company, and its chemical structure is shown in Figure 1A (Shanghai, China). The LPS and Donepezil were purchased from Sigma (Merck, Darmstadt, Germany). Nigericin was purchased from Yuanye Company (Yuanye Bio-Technology, Shanghai, China). The ROS detection kit was purchased from Wanlei Biotechnology (Wanlei Biotechnology, Shenyang, China). The hematoxylin–eosin staining (H&E) dye kits and some commercial kits including malondialdehyde (MDA), catalase (CAT) and superoxide dismutase (SOD) were purchased from Nanjing Jiancheng Bioengineering Research Institute (Nanjing Jiancheng Bioengineering Research Institute, Nanjing, China). Nissl staining solution and Bicinchoninic Acid Assay (BCA) protein concentration assay kits were purchased from Beyotime Biotechnology Co., Ltd. (Beyotime Biotechnology Co., Ltd, Shanghai, China). Interleukin-1β (IL-1β) and Tumor Necrosis Factor alpha (TNF-α) kits were purchased from Shanghai Yuanju Biotechnology Center (Shanghai Yuanju Biotechnology Center, Shanghai, China). The primary antibodies including Nrf2, HO-1, caspase-3, caspase-8, NLRP3, HMGB1, GSDMD, GSDMD-N, caspase-1, ASC, caspase-11, and GAPDH were purchased from Cell Signaling Technology (Danvers, MA, USA). The primary antibodies including BDNF, Tau, p-Tau, TLR4, MyD88, IKKβ, p-IKKβ, NF-κB p65, p-NF-κB p65, Bax, Bcl-2, and HSP70 were purchased from Wanlei Biotechnology (Wanlei Biotechnology, Shenyang, China).

### 2.2. Animal Treatment and Experimental Design

Male C57BL/6J mice (7 to 8 weeks, weighing 20~22 g) were obtained from LiaoNing Changsheng Experimental Animal Co., Ltd. with Certificate of Quality No. SCXK (Liao)2020-0001. The experimental animals were housed in cages under a 12 h light /12 h dark cycle (lights on at 8:00 a.m.), 60 ± 5% humidity, and a temperature of 25 ± 2 °C with free access to water and food. All experiments were in strict accordance with the Regulation on Administration of Experimental Animals of the Ministry of Science and Technology. All the steps of this study were approved by the Jilin Agricultural University Experimental Animal Ethics Committee (approval No. 20230619003).

Mice were randomly assigned to five groups, each consisting of eight mice, resulting in a total of forty mice. The normal group (group 1) was given saline, LPS group (0.5 mg/kg, intraperitoneal injection, group 2), LPS + maltol group (50 mg/kg, oral gavage, group 3), LPS + maltol group (100 mg/kg, oral gavage, group 4), and LPS + donepezil group (2 mg/kg, intraperitoneal injection, group 5) [14,20,21]. After 3 h of daily LPS injection stimulation, maltol and donepezil were administered to distinct groups. Euthanasia was conducted on the 14th day following the final administration via gradual carbon dioxide inhalation, and cervical dislocation was applied to confirm death. Subsequently, brain tissues and serum were obtained, rinsed in cold sterile deionized water under aseptic conditions, promptly frozen, and preserved at −80 °C for subsequent utilization.

### 2.3. Radial Arm Maze

Our experimental procedure was as described previously [22].

Mice were acclimatized to a phased fasting regimen with unlimited water the day prior to training. During this acclimatization period, the mice were allowed to explore the maze freely to familiarize themselves with the test environment, thereby minimizing stress; these exploratory attempts were not recorded. Training occurred over three consecutive days, commencing on day 8. Formal testing was conducted on days 11, 12, and 13, with one test administered each day. In each test group, the mice were positioned in the center of the maze and allowed to navigate freely for 5 min. At the conclusion of this period, the total distance traveled by the mice and the number of entries into the incorrect arm were recorded to assess their spatial memory capabilities.

### 2.4. Hematoxylin and Eosin (H&E) Staining

To evaluate the pathological changes of brain tissues from the hippocampus, including neuron loss, loose neuron structure, and decreased neuron density, H&E staining was employed. First, the brain tissues fixed in neutral buffered formalin were removed and subjected to conventional processing (dehydration with graded ethanol and permeabilization with xylene). Then, the brain tissues were embedded in paraffin. After the wax blocks were cooled, the blocks were cut into 10 μm thick slices on the rotary microtome [23] (Leica, RM2235, Solms, Germany). The sections were then stained with H&E staining kits and examined for pathological changes in the brain tissues by using a light microscope (Leica, DM750, Solms, Germany).

### 2.5. Nissl Staining

Coronal cryosections of 10 μm were stained with Nissl staining solution (Beyotime, C0117, Shanghai, China) for 5 min at 37 °C [24]. Then, samples were washed using 95% ethyl alcohol for 5 min and dried. Sections were then washed twice in xylene for 5 min. After being sealed with neutral balsam, the slides were observed under an optical microscope (Axio Image A2; Carl Zeiss, Oberkochen, Germany) by a blinded investigator.

### 2.6. Cell Culture and MTT Assay

HMC3 cells were provided by the Bethune School of Basic Medicine, Jilin University. HMC3 cells were cultured in Dulbecco’s Modified Eagle Medium (DMEM) supplemented with 20% Fetal Bovine Serum (FBS), 1% non-essential amino acids, and 1% penicillin–streptomycin at 37 °C in a water-jacketed CO_2_ incubator (Thermo, Rockford, IL, USA). After approximately 70–80% fusion, cells were inoculated into cell culture plates and further exposed to maltol (12.5, 25, and 50 μM) and LPS (100 ng/mL) + Nigericin (30 μM) for 24 h, except for the normal group. The cells were then washed with phosphate buffer solution (PBS) and measured for subsequent experiments.

The 3-(4,5-Dimethylthiazol-2-yl)-2,5-Diphenyltetrazolium Bromide (MTT) method was used to measure cell viability. Cells pre-cultured in DMEM for 24 h were exposed to different concentrations of maltol or LPS + Nig for 24 h. Then, 0.5 mg/mL MTT was added after incubation at 37 °C and 5% CO_2_ for 3.5 h. Dimethyl Sulfoxide (DMSO) was added to wells to dissolve dirty crystals. Cell viability was determined by specific absorption at 490 nm. All in vivo histological detections, protein extractions and quantitative analyses in this study were performed specifically in the mouse hippocampal region, which is the core brain area responsible for learning and memory function.

### 2.7. ROS Staining

HMC3 cells were treated with maltol, and 1 μM 2′,7′-dichlorodihydrofluorescein diacetate (DCFH-DA) was added at 37 °C for 30 min. Staining cells were washed with PBS twice before imaging [25]. Relative ROS fluorescence intensity was expressed as the percentage of the LPS induced group (Leica TCS SP8, Solms, Germany).

### 2.8. Flow Cytometry Analysis of HMC3 Cells

The apoptosis rate of HMC3 cells was detected by fluorescence method and iodine intensity (PI) flow cytometry [14]. The centrifuged cell precipitates were mixed in 5 μL and 200 μL of membrane-bound protein Annexin V/Fluorescein (V/FITC) binding buffer, respectively. The mixtures were incubated in the dark for 15 min, and then 200 μL of PBS was added for immediate flow cytometry analysis.

### 2.9. Immunofluorescent (IF) Staining

Simply put, for brain tissues from the hippocampus, 10 μm thick sections were performed with a stepwise elution treatment. Sections were then incubated with primary antibodies, including mouse anti-CD206 and rabbit anti-HSP70 were humidified overnight at 4 °C. Subsequently, SABC-Cy3-labeled secondary antibodies (1:300, Boster Biological Technology, Wuhan, China) were incubated at 37 °C for 30 min [26]. 4′,6-diamidino-2-phenylindole (DAPI) was applied for nuclear counterstaining to visualize all nuclei in brain tissue, and microglia were distinguished via specific molecular markers (Leica Microsystems, Wetzlar, Hesse, Germany).

### 2.10. Biochemical Analysis

The levels of IL-1β and TNF-α in serum samples and cell supernatants of each group were detected by ELISA kits [27]. The levels were detected according to the manufacturing company’s agreement, and the specific absorption was read at 450 nm on a reader (Bio-Rad, Hercules, CA, USA). The units of IL-1β and TNF-α are expressed as μg/mL.

The content of MDA and the activities of SOD and CAT in the hippocampus of the brain tissues were measured with an MDA assay kit (No. A003-1-2), SOD activity assay kit (No. A001-3-2) and CAT activity assay kit (No. A006-2-1), respectively. Detailed procedures followed manufacturer’s instructions.

### 2.11. Immunoprecipitation

HMC3 cells in culture plates were washed 3 times with clean PBS and then lysed with IP buffer and left to stand for 2 h. Co-immunoprecipitation (Co-IP) was then performed using the Pierce Co-immunoprecipitation Kit (Thermo Fisher Scientific, USA). Briefly, 50 µg of monoclonal TLR4 antibody was first covalently coupled to the magnetic bead antibody, then incubated with 200 mL of cell extract overnight at 4 °C, washed, and then pulled down and eluted from the antibody-bound protein complex. The eluted proteins were subjected to Western blotting to investigate the interaction between TLR4 and MyD88.

### 2.12. Immunohistochemistry (IHC) Staining

Briefly, the 10 μm thick sections were deparaffinized and rehydrated using a series of xylene and aqueous alcohol solutions. Following serum sealing, the sections were incubated overnight in a wet chamber at 4 °C with various antibodies, including polyclonal anti-NLRP3 (1:200) and anti-caspase1 (1:200). Afterward, the sections were incubated with secondary antibodies for 30 min. A substrate was then applied to the sections for 30 min, followed by diaminobenzidine (DAB) staining and hematoxylin counterstaining [28]. The positive staining was determined mainly by a brownish-yellow color in the cytoplasm or nucleus of the cells. An image was taken by light microscopy (Leica, DN750, Soles, and Germany).

### 2.13. Bioinformatics Analysis

Collection of AD microarray data set (GSE122063) from GEO data. GSE122063 was developed based on the GPL16699 platform, and this dataset included 56 AD patients and 44 control samples [29]. Using R version 4.2.1, the ggplot2 software package mapped the volcano and visualized the results of the difference analysis. After id conversion of the input molecular tables, GO and GSEA enrichment analyses were performed using the cluster *Profiler* package(v4.18.4).

### 2.14. Knockdown of HSP70 by Small Interfering RNA

The specific siRNAs targeting HSP70 were produced by Gene Pharma (Sangon Biotech (Shanghai) Co., Ltd., Shanghai, China). Following the manufacturer’s guidelines, HMC3 cells were transfected with a mixture of two pairs of HSP70-specific siRNA oligonucleotides (siHSP70) at a final concentration of 20 nM. The experimental protocols were as follows: HMC3 cells were transfected with HSP70-specific siRNA twice every two days using siRNAmate reagents. On day 5, the cell samples were treated with maltol and LPS + Nig. After 24 h of treatment, the cells were collected and lysed for subsequent analysis [22].

### 2.15. Western Blotting

The brain tissues from the hippocampus were ground with liquid nitrogen, and proteins were extracted promptly with RIPA lysate to determine the protein content [30]. The HMC3 cells were directly subjected to protein extraction and concentration determination. Sodium Dodecyl Sulfate–Polyacrylamide Gel Electrophoresis Gel (SDS-PAGE gel) was used to segregate protein samples, which were then electrophoretically transferred to Polyvinylidene Fluoride (PVDF) membranes, and the PVDF membrane was then closed with 5% skim milk for 2 h. Subsequently, the PVDF membranes were incubated overnight with primary antibodies against BDNF (1:1000), Tau (1:500), p-Tau (1:500), TLR4 (1:500), MyD88 (1:500), IKKβ (1:1000), p-IKKβ (1:1000), NF-κB p65 (1:1000), p-NF-κB p65 (1:1000), caspase-8 (1:1000); cleaved-caspase-8 (1:1000); caspase-3 (1:1000), cleaved-caspase-3 (1:1000), Bax (1:1000), Bcl-2 (1:1000), caspase-1 (1:1000), cleaved-caspase-1 (1:1000), caspase-11 (1:1000), cleaved-caspase-11 (1:1000), NLRP3 (1:500), HMGB1 (1:1000), GSDMD (1:1000), GSDMD-N (1:1000), ASC (1:1000), Nrf2(1:1000), HO-1(1:1000), GPX4 (1:1000), NOX4 (1:1000), SLC7A11 (1:1000) and HSP70 (1:1000) at 4 °C. GAPDH (1:1000) levels were measured as normal. The strips were then washed with tris buffered saline with tween-20 (TBST) and the secondary antibody (1:2000) was conjugated with the primary antibody for 2 h. Finally, a substrate (Pierce Chemical Co., Rockford, IL, USA) with Emitter-Coupled Logic (ECL) was used to capture signals. We used Image J 1.8.0.345 to quantify the bands (NIH, Bethesda, MD, USA).

### 2.16. Statistical Analysis

All data were recorded as mean ± standard deviation (Mean ± SD) and studied by one-way analysis of variance (ANOVA) and Bonferroni post hoc test. The statistical graph data were generated through GraphPad Prism 8.0.2 software (GraphPad Software, Inc., San Diego, CA, USA). In all cases, *p* < 0.001, *p* < 0.01 or *p* < 0.05 was significant.

## 3. Results

### 3.1. Maltol Improves Memory Loss and Ameliorated AD-like Symptoms In Vivo

Mice were stimulated by LPS for 14 consecutive days and then executed. The experimental design is shown in (Figure 1B). To test whether maltol can improve memory loss in mice, we trained the mice for three consecutive days using an eight-arm maze. In the formal test, we recorded the incubation period and the total distance of movement to assess the memory ability of the subject mice (Figure 1C,D). In comparison to the normal group, mice in the model group exhibited a significant increase in both latency time and total distance traveled, suggesting a reduction in memory capacity. Following the administration of maltol (50 mg/kg and 100 mg/kg) and donepezil (2 mg/kg), a notable enhancement in the memory capacity of the mice was observed relative to the model group.

Memory loss will inevitably lead to different degrees of pathological damage in the brain. Therefore, we further confirmed the improvement of maltol on brain memory impairment in mice by H&E and Nissl staining. The results from H&E staining showed that the structure of hippocampal neurons in the model group was loose, and the neuronal density was significantly decreased (*p* < 0.01) in the CA3 region. Quantification was performed on six coronal sections per mouse, eight mice per group (Figure 1E). Maltol and donepezil significantly alleviated injury and increased neuronal density (*p* < 0.05, *p* < 0.01). Additionally, changes in the number of Nissl bodies are commonly used to determine the extent of nerve cell damage. In the normal group, the neuronal cells in the CA3 area of the hippocampus were arranged, the cell morphology was round, and the outline of the cytosol and the Nissl bodies were clear, while in the model group, the neurons in the CA3 area were arranged in a disordered manner, and the number of Nissl bodies was reduced. At the same time, maltol and donepezil effectively alleviated the above pathology.

Finally, we examined the expression of BDNF and Tau proteins by Western blotting. The expression level of BDNF protein was significantly decreased, and the expression level of Tau protein was also significantly decreased after abnormal phosphorylation, which was significantly reversed after maltol and donepezil administration (*p* < 0.05) (Figure 1F,G). These results suggested that maltol may have a protective effect against memory loss.

### 3.2. Maltol Reduces LPS-Induced Apoptosis In Vitro and In Vivo

**Figure 2:**
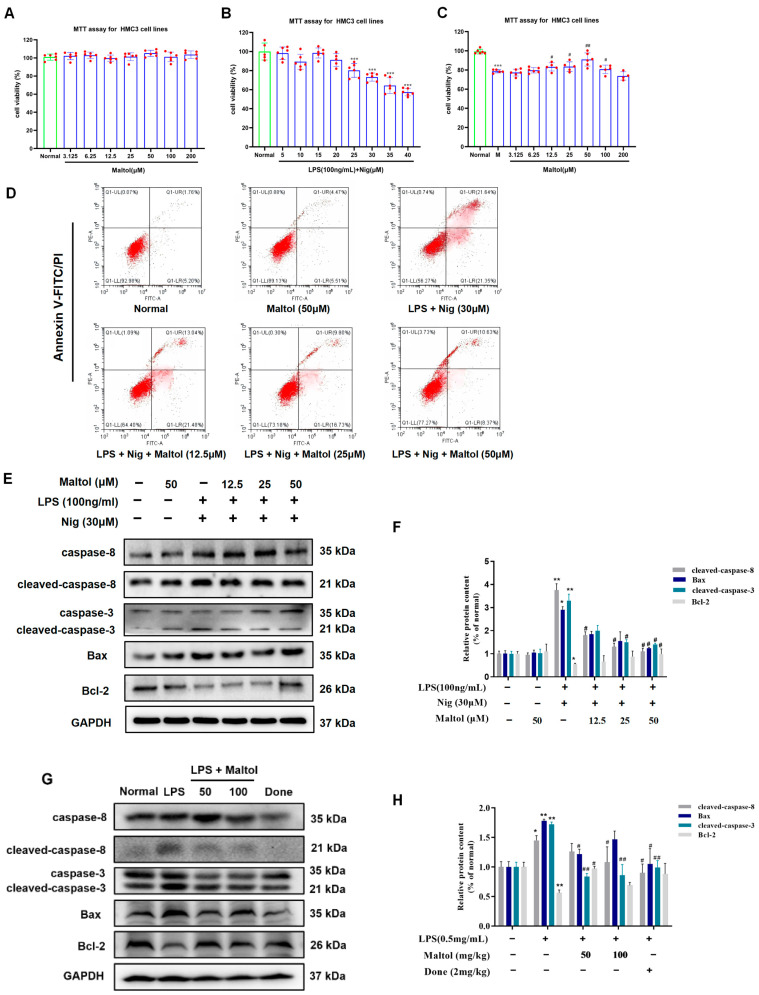
Maltol reduced LPS-induced apoptosis in vitro and in vivo. (**A**) Test of maltol on cytotoxicity in HMC3 cells. (**B**) HMC3 cells injury induced by LPS + Nig. (**C**) Effect of maltol on damage induced by LPS + Nig in HMC3 cells. (**D**) Cell flow cytometric analysis. (**E**,**F**) Western blot analysis of caspase-3, cleaved-caspase-3 (cl-caspase-3), caspase-8, cleaved-caspase-8 (cl-caspase-8), Bax, and Bcl-2 in HMC3 cells. (**G**,**H**) Western blot analysis of caspase-8, cleaved-caspase-8 (cl-caspase-8), caspase-3, cleaved-caspase-3 (cl-caspase-3), Bax, and Bcl-2 in brain tissues. Data are expressed as Mean ± S.D. (n = 8 in each group). * *p* < 0.05, ** *p* < 0.01, *** *p* < 0.001 vs. normal group; # *p* < 0.05, ## *p* < 0.01, vs. LPS group.

To examine the protective effect of maltol against LPS-induced apoptosis in neuronal cells, we utilized the human microglia HMC3 cell line to establish a model of neuronal pyroptosis induced by LPS and Nigericin. After detecting the toxicity of maltol in a certain dose range (Figure 2A), the optimal dose of LPS and Nig combined to model the HMC3 cell line was determined, and it was found that the cell viability value decreased to 78% after 24 h of combined LPS (100 ng/mL) and Nig (30 μM) stimulation (*p* < 0.05, *p* < 0.01), which can be used for follow-up experiments (Figure 2B). The protective effect of maltol reached saturation at 50 μM, and higher concentration failed to further improve cell viability. Excessively high maltol concentration may induce mild metabolic stress, leading to a stable dose–effect plateau. Then, to evaluate the protective effect of maltol on HMC3 cells induced by LPS and Nig, we first pre-protected HMC3 cells with maltol (3.125, 6.25, 12.5, 25, 50, 100, and 200 μM), and then the viability of HMC3 cells was measured in a dose-dependent manner 24 h after exposure to LPS (100 ng/mL) and Nig (30 μM). The results showed that maltol at concentrations from 12.5 to 50 μM significantly improved the cells compared with the model group (*p* < 0.05, *p* < 0.01) (Figure 2C).

To evaluate the anti-apoptotic effect of maltol in HMC3 cells, we detected apoptosis using flow cytometry. The apoptosis rate in the model group was 21.64% compared to the normal group, indicating that LPS and Nig induced apoptosis in HMC3 cells. The flow cytometry results showed a dose-dependent decrease in apoptosis rate after maltol treatment (*p* < 0.05, *p* < 0.01) (Figure 2D). Subsequently, apoptosis-related proteins were detected in HMC3 cells and brain tissues, respectively, and it was found that the expression of cleaved-caspase-8, cleaved caspase-3 and Bax proteins increased in the model group; the expression of Bcl-2 protein decreased; and maltol and donepezil could reverse these trends (*p* < 0.05) (Figure 2E–H). These results suggested that maltol may have an antagonistic effect on LPS-induced apoptosis in vitro and in vivo.

These also indicate that the decreased cell viability of HMC3 cells induced by LPS is not regulated by a single cell death pattern. Combined with the experimental results in this study, the decline in cell viability was co-mediated by apoptosis and microglial pyroptosis. In addition, consistent with the findings in Section 3.5, ferroptosis also contributed to LPS-triggered cell injury and viability reduction. Collectively, apoptosis, pyroptosis and ferroptosis jointly participate in LPS-mediated microglial cell damage, and maltol can alleviate such cell injury by suppressing all three types of cell death.

### 3.3. Maltol Inhibited LPS-Induced Neuroinflammation In Vitro and In Vivo

**Figure 3:**
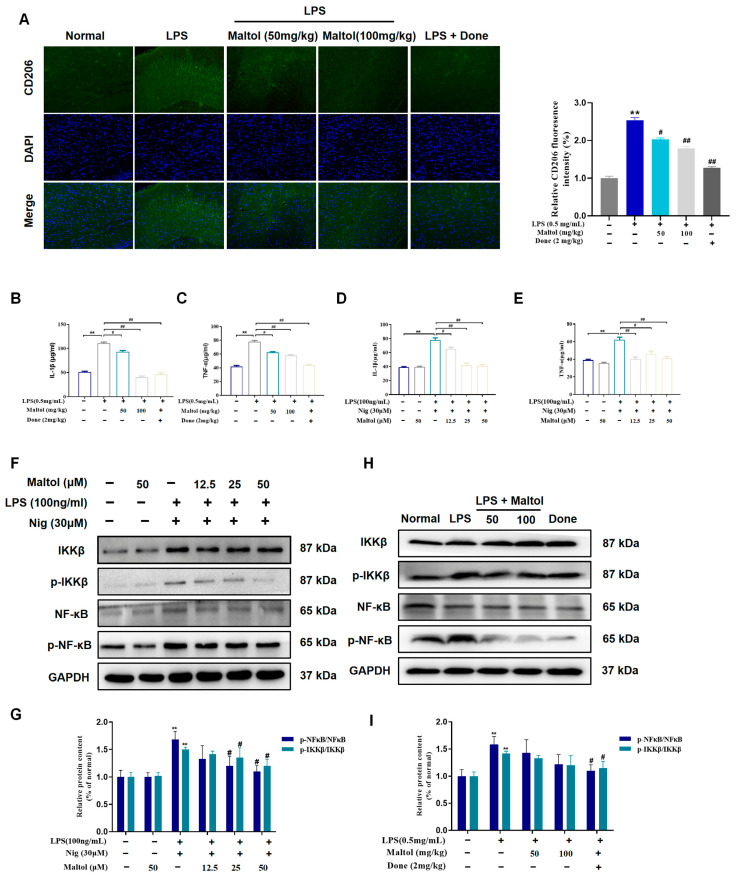
Maltol inhibited LPS-induced neuroinflammation in vitro and in vivo. (**A**) IF staining of CD206 in brain tissues. Relative CD206 fluorescence intensity was quantified in 6 sections per mouse, 8 mice per group, 5 random fields per section. (**B**,**C**) Analysis of IL-1β and TNF-α levels in brain tissues. (**D**,**E**) Analysis of IL-1β and TNF-α levels in HMC3 cells. (**F**,**G**) The protein expressions of IKKβ, p-IKKβ, NF-κB p65, and p-NF-κB p-p65 in HMC3 cells. (**H**,**I**) The protein expressions of IKKβ, p-IKKβ, NF-κB p65, and p-NF-κB p-p65 in brain tissues. Quantification of relative protein contents were performed by densitometric analysis. Data are expressed as Mean ± S.D. (n = 8 in each group). ** *p* < 0.01 vs. normal group; # *p* < 0.05, ## *p* < 0.01 vs. LPS group.

As a marker of the M2 type of macrophages, CD206 was detected in the cortical area of the hippocampus of the brain tissues by immunofluorescence (IF) method (Figure 3A). Compared with the normal group, the fluorescence intensity of CD206 in the model group was significantly increased, indicating that LPS could induce the activation of microglia, and maltol and donepezil could reverse this phenomenon to a certain extent. Subsequently, we detected the levels of inflammatory factors IL-1β and TNF-α in HMC3 cells and brain tissues, respectively. The levels of IL-1β and TNF-α were significantly increased in the model group, and maltol and donepezil could inhibit the production of inflammatory factors by inhibiting the activation of microglia (*p* < 0.05, *p* < 0.01) (Figure 3B–E).

We next examined inflammatory response-associated proteins in HMC3 cells and brain tissues by Western blotting. Compared with the normal group, LPS increased the expression levels of p-NF-κB p65 and p-IKKβ, which were alleviated after maltol and donepezil treatment (*p* < 0.05, *p* < 0.01) (Figure 3F–I). These results suggested that maltol treatment may play an anti-inflammatory role by inhibiting NF-κB p65-mediated neuroinflammation.

### 3.4. Maltol Alleviates LPS-Induced Neuroinflammation and Pyroptosis by Inhibiting TLR4/MyD88 Binding

**Figure 4:**
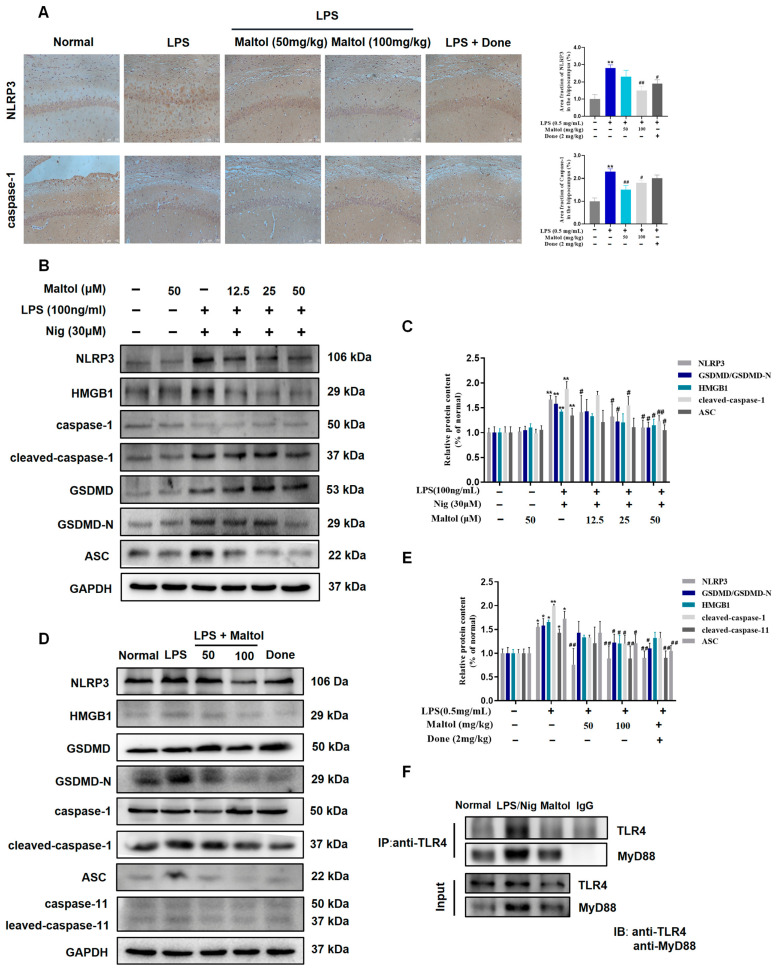
Maltol alleviated LPS-induced neuroinflammation and pyroptosis by inhibiting TLR4/MyD88 binding. (**A**) IHC analysis of NLRP3 and caspase-1 protein of maltol on LPS-induced cell injury. The relative areas of NLRP3 and caspase-1 in 6 sections of each mouse were quantitatively measured. There were 8 mice in each group, and 5 random regions were selected for each section. (**B**,**C**) The protein expressions of NLRP3, HMGB1, GSDMD, GSDMD-N, caspase-1, cleaved-caspase-1 (cl-caspase-1), and ASC in HMC3 cells. (**D**,**E**) The protein expressions of NLRP3, HMGB1, GSDMD, GSDMD-N, caspase-1, cleaved-caspase-1 (cl-caspase-1), caspase-11, cleaved-caspase-11 (cl-caspase-11), and ASC in brain tissues. (**F**) Co-IP analysis of TLR4 and MyD88 protein. Data are expressed as Mean ± S.D. (n = 8 in each group). * *p* < 0.05, ** *p* < 0.01 vs. normal group; # *p* < 0.05, ## *p* < 0.01 vs. LPS group.

To investigate the role of maltol in LPS + Nig-induced pyroptosis and its mechanism of action, we verified it by immunohistochemistry (IHC) and Western blot. The IHC results indicated a significant increase in brown positive particles for NLRP3 and caspase-1 in the CA1 region of the model group compared to the normal group. This finding suggests the activation of the NLRP3 inflammasome, which was subsequently reversed following treatment with maltol and donepezil (Figure 4A). We simultaneously examined the expression of classical pyroptosis pathway-associated proteins induced by NLPR3 activation and non-classical pyroptosis pathway proteins mediated by caspase-11. As shown in (Figure 4B,C), compared with the normal group, the expression levels of NLRP3, HMGB1, cl-caspase-1, GSDMD/GSDMD-N, ASC and cl-caspase-11 in the model group were increased in vivo, which was significantly downregulated after maltol and donepezil treatment (*p* < 0.05, *p* < 0.01). In addition, the expression levels of NLRP3, HMGB1, cl-caspase-1 and GSDMD/GSDMD-N in HMC3 cells in the model group were higher than those in normal group, and these phenomena were eliminated to a certain extent after maltol treatment (*p* < 0.05, *p* < 0.01) (Figure 4D,E).

We next examined the binding of TLR4 and MyD88 in HMC3 cells. Co-IP results showed that in the IP model group, the expression levels of TLR4 and MyD88 were elevated, which was alleviated by maltol treatment (*p* < 0.01) (Figure 4F). In the input control LPS + Nig group, the expression levels of TLR4 and MyD88 were elevated and decreased after maltol treatment. It is suggested that maltol may alleviate NF-κB p65 activation by promoting TLR4/MyD88 dissociation.

### 3.5. Maltol Ameliorates Oxidative Stress and Ferroptosis In Vitro and In Vivo

**Figure 5:**
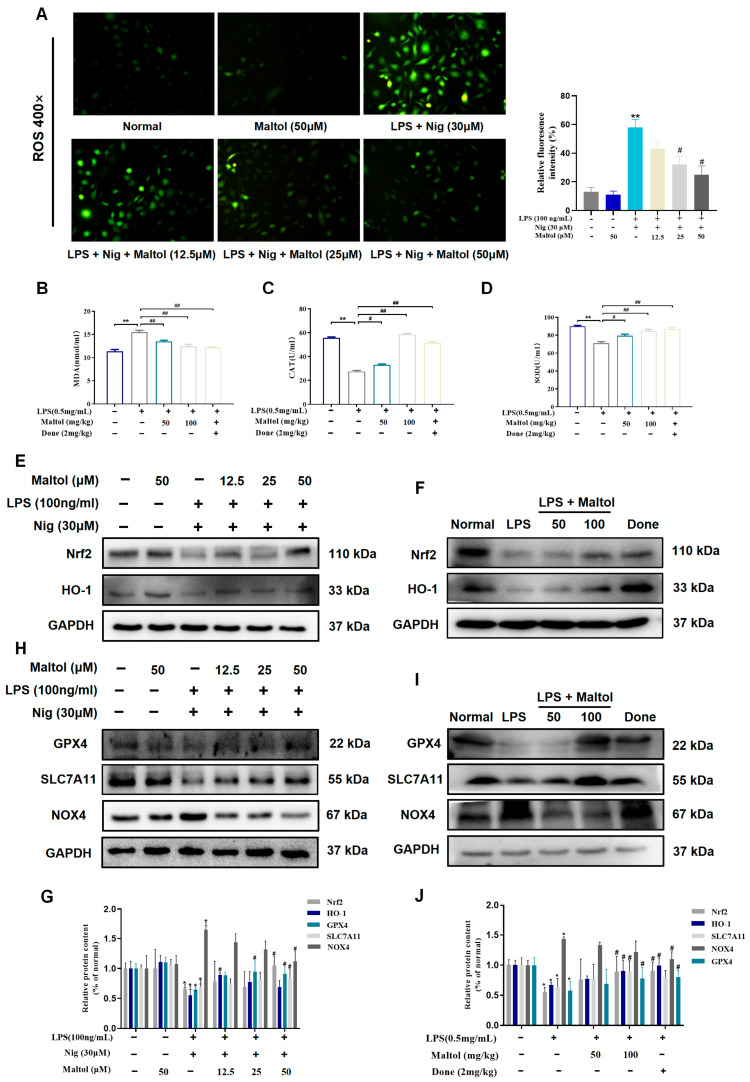
Maltol ameliorated oxidative stress and ferroptosis in vitro and in vivo. (**A**) Intracellular ROS accumulation in HMC3 cells was observed by DCFH-DA fluorescent staining (magnification, 400×); all in vivo ROS detections were conducted in the hippocampal CA3 region. Relative ROS fluorescence intensity was quantified in 6 sections per mouse, 8 mice per group, 5 random fields per section. The levels of (**B**) MDA, (**C**) CAT, and (**D**) SOD in the brain tissues. (**E**,**F**) Expression of oxidative stress-related proteins Nrf2 and HO-1 in HMC3 cells and brain tissues. (**H**,**I**) Expression of ferroptosis proteins GPX4, SLC7A11, and NOX4 in HMC3 cells and brain tissues. (**G**–**J**) Quantification of relative protein contents were performed by densitometric analysis in HMC3 cells and brain tissues. Data are expressed as Mean ± S.D. (n = 8 in each group). * *p* < 0.05, ** *p* < 0.01 vs. normal group; # *p* < 0.05, ## *p* < 0.01 vs. LPS group.

To analyze the relationship between maltol and oxidative stress, we used fluorescent staining, biochemical analysis, and Western blot analysis to analyze the antioxidant effect of maltol. Fluorescent staining analysis showed that LPS significantly increased ROS overproduction in HMC3 cells, while ROS fluorescence intensity was quantitatively analyzed in five random fields per well, and normalized to the LPS group. Maltol significantly reduced ROS overproduction (*p* < 0.01) (Figure 5A). Meanwhile, in model group, the levels of CAT and SOD activities were significantly decreased, while the activity levels of MDA were significantly increased in vivo (*p* < 0.05) (Figure 5B–D). Western blotting detected the expression levels of oxidative stress-related proteins (Nrf2 and HO-1), and the results showed that maltol and donepezil could effectively relieve LPS-induced oxidative stress both in vitro and in vivo (*p* < 0.05, *p* < 0.01) (Figure 5E–G).

We then analyzed whether maltol could ameliorate oxidative stress and ferroptosis through the Nrf2/GPX4 axis. In vitro and in vivo results consistently showed that GPX4 and SLC7A11 protein expression was significantly reduced in the model group compared with the normal group, and NOX4 protein expression was increased, which were reversed after maltol treatment (*p* < 0.05, *p* < 0.01) (Figure 5H–J). Accumulating evidence indicates that exposure to lipopolysaccharide (LPS) can induce ferroptosis in both cultured HMC3 cells and hippocampal tissues of mice. Notably, the ferroptosis induced by LPS in vivo primarily occurs in the microglial cells of the hippocampus, suggesting an indirect effect on neurons. In this study, all tissue sampling and detection of ferroptosis-related molecular indicators were conducted specifically in the hippocampal region. Maltol intervention effectively reversed LPS-induced alterations in ferroptosis-associated proteins, reduced the accumulation of reactive oxygen species, and ultimately mitigated ferroptotic injury in hippocampal microglia.

### 3.6. Maltol Enhances the Expression of HSP70 In Vitro and In Vivo

**Figure 6:**
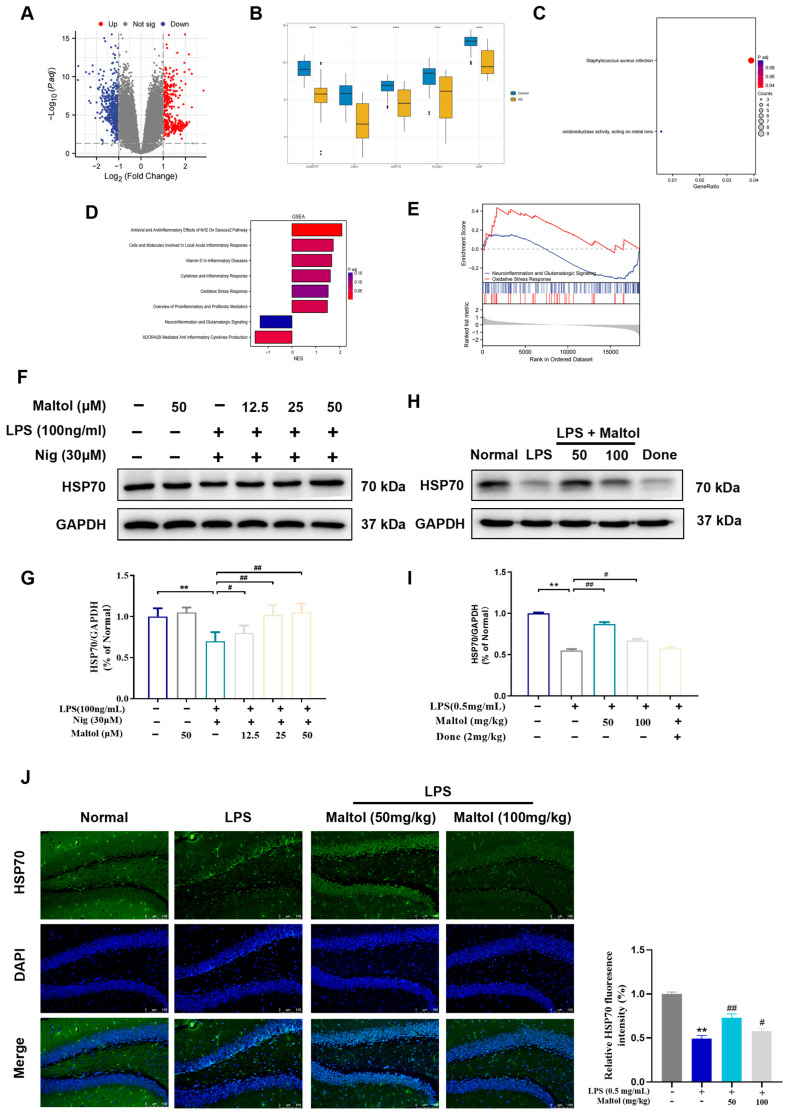
Maltol enhanced the expression of HSP70 in vitro and in vivo. (**A**) Differential gene volcano map of AD in GSE122063 database. (**B**) HSP70 differential gene content. (**C**) GSEA data analysis. (**D**,**E**) Expression of HSP70 in HMC3 cells and brain tissues. (**F**,**G**) Quantification of HSP70 protein contents were performed by densitometric analysis in HMC3 cells and brain tissues. (**H**) IF analysis of HSP70 in brain tissues. Relative HSP70 fluorescence intensity was quantified in 6 sections per mouse, 8 mice per group, 5 random fields per section. Data are expressed as Mean ± S.D. (n = 8 in each group). ** *p* < 0.01 vs. normal group; # *p* < 0.05, ## *p* < 0.01 vs. LPS group.

The results showed that 341 genes were upregulated and 559 genes were downregulated (*p* < 0.05) (Figure 6A). The amount of HSP70 gene difference between the AD and healthy group is shown in Figure 6B. Gene Set Enrichment Analysis (GSEA) showed that HSP70 gene was related to neuroinflammation, the glutamatergic signaling pathway and the oxidative stress response signaling pathway (Figure 6C).

To determine whether maltol could affect HSP70 expression in mice and HMC3 cells, levels of HSP70 were measured by Western blot and IF. Western blot results showed that compared with the normal group, the expression level of HSP70 in the model groups was significantly decreased, while maltol treatment significantly increased the expression level of HSP70 in HMC3 cells (*p* < 0.05, *p* < 0.01) (Figure 6D,E). Compared with the normal group, the level of HSP70 protein in the model group was significantly reduced and recovered after treatment with maltol and donepezil in vivo (*p* < 0.05, *p* < 0.01) (Figure 6F,G). The same results were obtained by IF analysis of HSP70 protein in brain tissues (*p* < 0.05, *p* < 0.01) (Figure 6H). These results suggested that maltol may play a key role in neuroprotection by at least upregulating HSP70 expression.

### 3.7. Maltol Blocks the Inflammation and Microglial Pyroptosis by Increasing the Expression Level of HSP70

**Figure 7:**
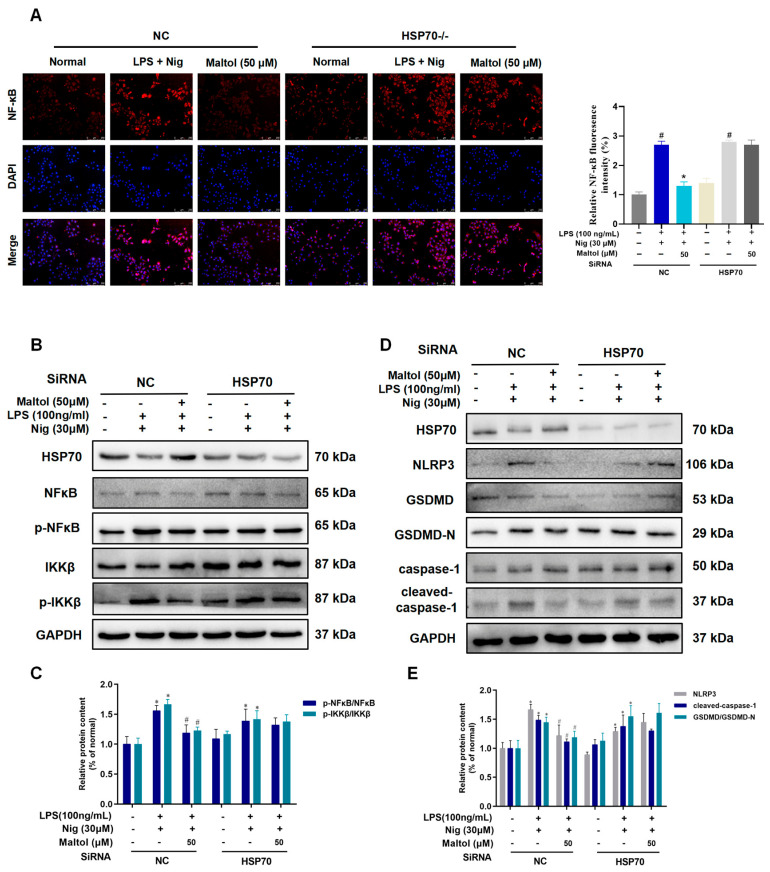
Maltol blocked the inflammation and microglial pyroptosis by increasing the expression level of HSP70. (**A**) Immunofluorescence staining analysis of NF-κB p65 in HMC3 cells. (**B**,**C**) Western blot analysis of inflammation-related proteins after treatment. (**D**,**E**) Western blot analysis of pyroptosis-related proteins after treatment. Data are expressed as Mean ± S.D. (n = 8 in each group). * *p* < 0.05, vs. normal group; # *p* < 0.05 vs. LPS group.

To investigate whether maltol could play a neuroprotective role through HSP70, we silenced HSP70 gene in HMC3 cells. The siRNA of HSP70 was added into the culture medium. HMC3 cells were treated with siRNA (20 nM) for 48 h, the expression of HSP70 protein was downregulated by 80%. The IF analysis results showed that compared with the NC group, the NF-κB p65 activation induced by LPS + Nig was not relieved in the HSP70-/- group after maltol treatment (Figure 7A). Meanwhile, Western blot results also showed that in the HSP70-/- group, maltol treatment did not inhibit LPS + Nig-induced phosphorylation of NF-κB p65 and IKKβ (Figure 7B,C). Similarly, maltol treatment did not inhibit the expression of NLRP3, GSDMD/GSDMD-N, or cl-caspase-1 (Figure 7D,E). These results suggested that HSP70 may be a key target for maltol to play a role in improving LPS-mediated inflammation and pyroptosis.

## 4. Discussion

In recent years, the significant role of neuroinflammation in Alzheimer’s disease (AD) has garnered increasing attention. Research indicates that in the early stages of AD, neurons producing Tau may trigger neuroinflammation by recruiting microglia and promoting the release of pro-inflammatory cytokines and chemokines, thereby exacerbating the progression of the disease [31]. Prolonged neuroinflammation inevitably damages neurons involved in learning and memory and can precipitate premature aging and central nervous system disorders. Therefore, preventing or interrupting neuroinflammation is critical, and identifying safe, effective therapeutics to ameliorate it is urgent. Our previous studies have shown that maltol, a flavoring agent, can delay brain aging [13] and can effectively improve the intestinal inflammatory response induced by cisplatin [16]. For these reasons, we hypothesize that maltol may have the potential to be an effective agent for ameliorating LPS-induced neuroinflammation.

**Figure 8:**
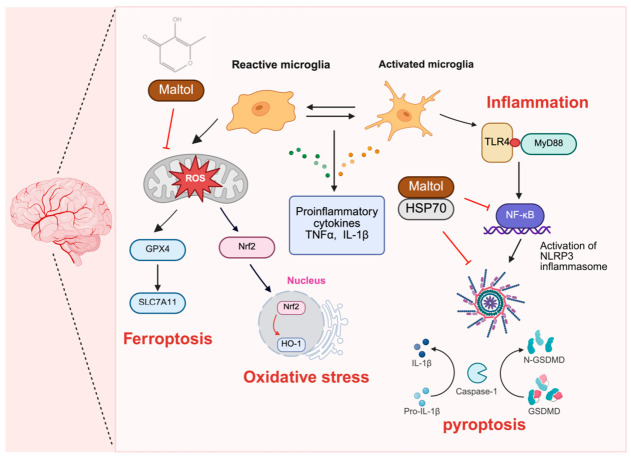
Molecular mechanism of maltol for ameliorating LPS-induced neuroinflammation and pyroptosis of microglia by increasing the expression level of HSP70 protein (created by Biorender.com).

In this study, experimental models of LPS-induced neuroinflammation in vitro and in vivo were established to investigate the ameliorative effect of maltol for the first time (Figure 8). The findings indicated that maltol enhanced mice’s memory/learning capacity, suppressed HMC3 cell apoptosis, and mitigated ferroptosis induced by oxidative stress (*p* < 0.05, *p* < 0.01). The inflammation and pyroptosis of HMC3 cells were relieved by upregulating HSP70 expression (*p* < 0.05, *p* < 0.01). Following 14 days of consecutive LPS injections, the behavioral test results revealed increased food-seeking time and total distance traveled in the model group. Pathological staining clearly displayed hippocampal tissue damage and necrosis. Reduced expression levels of BDNF and Tau proteins were also evident. These features collectively validate the successful establishment of the neuroinflammatory model [32]. After maltol and donepezil treatment, memory ability, pathological staining, BDNF and Tau protein levels of mice were improved to varying degrees (*p* < 0.05, *p* < 0.01), which further confirmed the improvement of maltol on memory.

Apoptosis is crucial for maintaining normal physiological processes, with the endogenous apoptosis pathway involving downstream caspase-3 activation by the Bcl-2 protein family being the most extensively studied [33]. Moreover, the exogenous apoptotic pathway mediated by caspase-8 has garnered significant attention recently for its role in initiating caspase activation [34]. Prior research has demonstrated that in macrophages, caspase-8 can utilize scaffold proteins to engage in NLRP3 inflammasome activation triggered by dsRNA. Its activity is also essential for NF-κB p65 activation and cytokine secretion [35]. Thus, we assessed the expression levels of Bcl-2 family-associated proteins (Bax, Bcl-2, caspase-3) and caspase-8. Our findings revealed that maltol effectively reversed LPS-induced apoptosis by reducing Bax, caspase-3, and caspase-8 levels, while elevating Bcl-2 levels both in vitro and in vivo (*p* < 0.05, *p* < 0.01). Flow cytometry results further confirmed these outcomes in HMC3 cells.

Neuroinflammation is frequently regarded as a primary contributor to AD [36]. Microglia, the principal resident immune cells in the central nervous system, are pivotal in the initiation and activation of neuroinflammation. Numerous studies have demonstrated that microglial activation leads to the release of elevated levels of pro-inflammatory factors and cytotoxic substances, which in turn cause neuronal dysfunction and cell death [37]. In this study, the IF analysis results showed that the fluorescence level of CD206 protein in HMC3 cells was significantly increased after LPS stimulation, indicating that the cells had been induced to activate (*p* < 0.05). The results of the levels of pro-inflammatory factors showed that activated HMC3 cells increase the release of TNF-α and IL-1β, and aggravated the occurrence of neuroinflammation, which was consistent with the results reported in the literature [37,38]. However, these phenomena were relieved after maltol treatment in vitro and in vivo, respectively. The results of Western blot analysis also verified that maltol could reverse the activation of NF-κB p65 and IKKβ protein induced by LPS and exert anti-inflammatory effects to some extent (*p* < 0.05, *p* < 0.01).

Pyroptosis, a type of programmed cell death facilitated by GSDMD, is distinguished by cell swelling and the discharge of cellular contents, triggering heightened inflammatory and immune reactions [39]. Research indicates that pyroptosis primarily relies on the stimulation of caspase proteins by the NLRP3 inflammasome to induce diverse physiological responses [40,41]. It can be categorized into classical pathways instigated by caspase-1, or non-classical pathways initiated by caspase-11 or caspase-4/5, depending on the specific pathway [42]. In vitro studies, we used nigericin, a NLRP3 activator, in combination with LPS to induce a pyroptosis model of HMC3 cells. Western blot and IHC analysis results showed that maltol could effectively reduce the activation of the classical pathway of caspase-1 caused by NLPR3 activation and the non-classical pathway mediated by caspase-11 activation compared with the model group. TLR4, as an important pattern recognition receptor for dangerous and pathogen-associated molecular patterns (DAMPs and PAMPs), has been shown to play a key role in LPS-induced caspase-4/5/11-dependent pyroptosis [43]. Silveira LS et al. reported that TLR4 can enhance the transcription of the NLRP3 component by binding to MyD88, which activates downstream NF-κB p65 and subsequently regulates pyroptosis through an inflammatory response [44,45]. Our findings also demonstrated that maltol inhibits LPS-induced activation of NF-κB p65 and NLRP3 by disrupting TLR4/MyD88 binding, thereby mitigating inflammation and pyroptosis. (*p* < 0.05, *p* < 0.01).

Oxidative stress, characterized by the imbalance between oxidation and antioxidation within the body, triggers the inflammatory infiltration of neutrophils and the generation of various oxidative intermediates [46]. This phenomenon affects virtually all organs and is particularly linked to neurodegenerative conditions like Alzheimer’s disease and Parkinson’s disease [47]. Research indicates that elevated levels of reactive oxygen species (ROS), a hallmark of oxidative stress, play a significant role in promoting ferroptosis [48]. In line with the existing literature, our experimental findings demonstrated a substantial increase in ROS production in HMC3 cells induced by LPS/Nig. Subsequent analysis included the evaluation of oxidative stress-related proteins (Nrf2 and HO-1) and the activities of antioxidation-related proteins (SOD and CAT). These findings indicate that maltol may exert antioxidant effects (*p* < 0.05, *p* < 0.01). Malondialdehyde (MDA) is a significant product of lipid peroxidation, reflecting its extent. Analysis of MDA levels revealed that maltol mitigated the rapid increase in MDA induced by LPS. Evaluation of ferroptosis-associated proteins demonstrated that maltol administration upregulated the protein levels of GPX4 and SLC7A11 while downregulating NOX4 protein expression (*p* < 0.05, *p* < 0.01). These results suggest that maltol might mitigate ferroptosis triggered by LPS-induced oxidative stress.

To further evaluate the potential role of maltol in improving LPS-induced AD-related neuroinflammation through deeper targeting mechanisms, we used GEO database to identify differential genes in AD disease. After GSEA enrichment analysis, it was found that HSP70 was highly correlated with oxidative stress and neuroinflammatory response. Choi H. et al. found that acetylated Tau in animal and organoid models of AD recruits the chaperone protein HSP70 to alter the Tau interaction set to degrade Tau [49]. In short, HSP70 plays a crucial role in Tau depolymerization and refolding. Camila Tiefensee Ribeiro et al. found that the neuroprotective effect of HSP70 may regulate the inflammatory response in microglia by inhibiting the activation of inducible NOS (iNOS) and NF-κB p65 [50]. Therefore, it is reasonable to assume that maltol maybe exert a series of neuroprotective effects through the HSP70 gene. In this study, the results of detecting HSP70 expression in vitro and in vivo showed that maltol could effectively improve the expression level of HSP70, which was basically consistent with the speculation. To further confirm our conjecture, we performed gene silencing for HSP70 in HMC3 cells to determine whether maltol had the potential to block various undesirable signaling pathways through HSP70 targets. The results showed that maltol treatment did not alleviate LPS/Nig exposure-induced NF-κB p65/NLRP3-mediated neuroinflammation and pyroptosis. These results suggested that maltol may exert anti-inflammatory and anti-pyroptosis effects on HMC3 cells by increasing the expression level of HSP70 protein. Since pyroptosis was only detected in HMC3 microglia rather than in neurons in the present study, maltol protected neurons mainly by inhibiting microglial pyroptosis and neuroinflammation, thereby indirectly reducing secondary neuronal injury and apoptosis.

## 5. Conclusions

In conclusion, this study established LPS-induced neuroinflammatory Alzheimer’s disease models in vitro and in vivo to explore the ameliorative effects of maltol on microglia in signaling pathways such as inflammation, pyroptosis, and oxidative stress. Differential gene HSP70 was screened out in GEO database, and gene silencing was used to investigate the effect of maltol on inhibiting inflammation and pyroptosis by increasing the expression level of HSP70. This may provide a new direction and theoretical basis for finding new drugs for the treatment of AD. Indeed, the downregulation of Tau and the modulation of BDNF by maltol are unlikely to be regulated solely by elevated HSP70. Other potential mechanisms, including anti-neuroinflammation, antioxidant stress, and the regulation of related upstream signaling pathways, may also jointly participate in mediating the changes of these two proteins. Further research is still needed to explore more underlying regulatory networks.

In future work, we will further explore how HSP70 can be used as one of the important chaperones to improve the unstructured region binding of incomplete or misfolded proteins caused by neuroinflammation.

## Data Availability

The original contributions presented in this study are included in the article. Further inquiries can be directed to the corresponding authors.

## Abbreviations

AD: Alzheimer’s disease; maltol: 3-hydroxy-2-methyl-4-pyrone; LPS: lipopolysaccharide; Nig: nigericin; ROS: reactive oxygen species; MDA: malondialdehyde; CAT: catalase; SOD: superoxide dismutase; MTT: methyl–thiazolyl–tetrazolium; H&E: hematoxylin and eosin.
